# Supplementary figures and images for: Multiplex vs. singleplex assay for the simultaneous identification of the three components of avian malaria vector-borne disease by DNA metabarcoding
Source: PeerJ. 2025 Mar 18;13:e19107. doi: 10.7717/peerj.19107 (PMC11927560; doi:10.7717/peerj.19107)

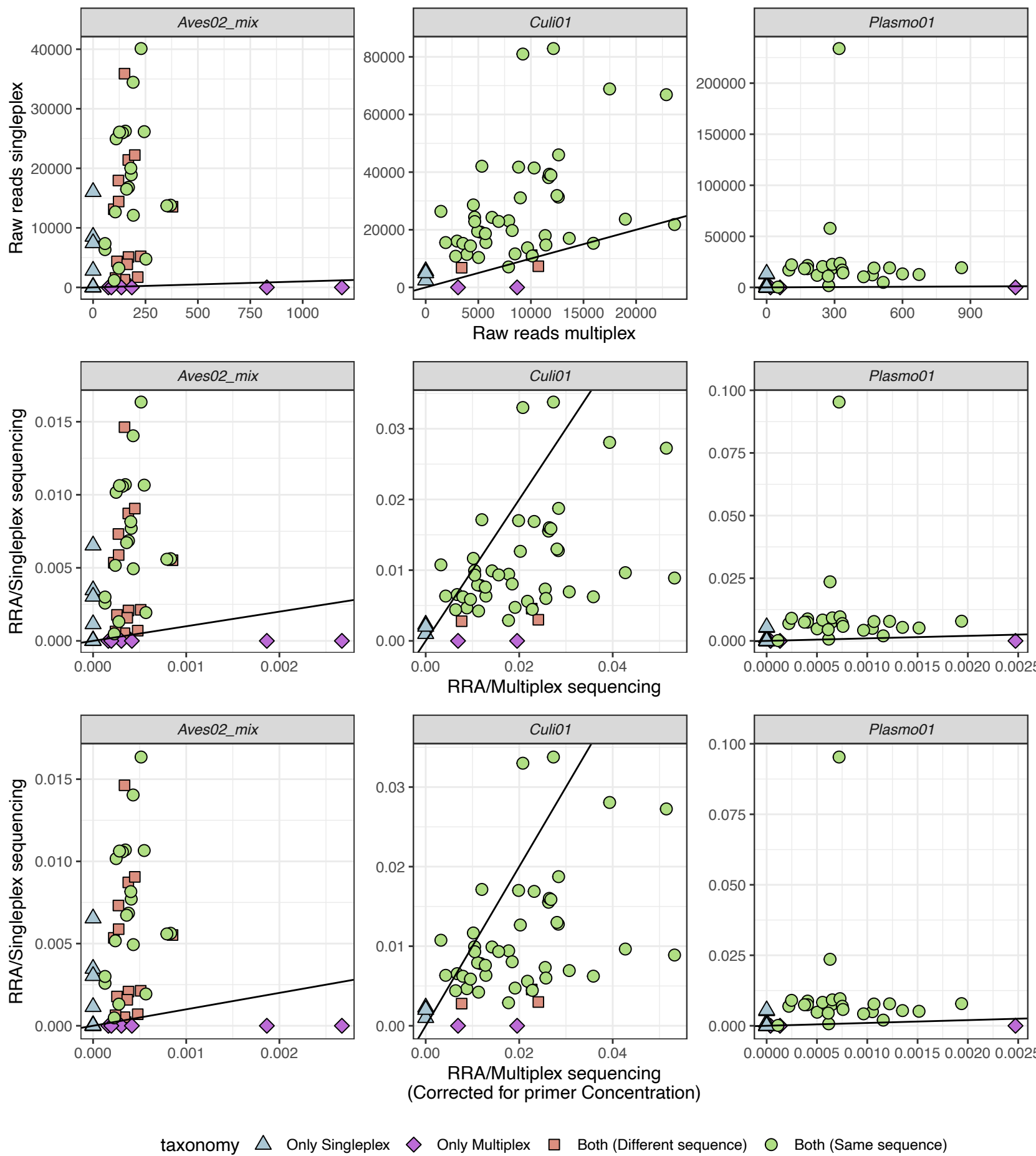

Supplement: Figure S1 — (B) Comparison between the RRA obtained using singleplex vs. multiplex for each particular sample and primer pair. (C) Comparison between the RRA obtained using singleplex vs. multiplex for each particular sample and primer pair corrected for the relative primer concentration used in the multiplex approach (see Materials and Methods). [file peerj-13-19107-s001.pdf]
