# Supplementary material for: Multiplex vs. singleplex assay for the simultaneous identification of the three components of avian malaria vector-borne disease by DNA metabarcoding
Source: PeerJ. 2025 Mar 18;13:e19107. doi: 10.7717/peerj.19107 (PMC11927560; doi:10.7717/peerj.19107)
Supplement: Table S1 [file peerj-13-19107-s002.docx]

**Table S1**. Bird species and *Plasmodium* mtDNA cytochrome *b* lineages[(Bensch et al., 2009)](https://www.zotero.org/google-docs/?4wyGyK) tested in preliminary PCRs with *Aves02* and *Plas01* primers, respectively.

| **Bird species tested** | **Common name** | ***Plasmodium* lineages tested** |
| --- | --- | --- |
| *Accipiter gentilis* | Eurasian goshawk | AEDVEX01 |
|  |  |  |
| *Anseranas semipalmata* | Magpie goose | AFR046 |
|  |  |  |
| *Apus apus* | Common swift | AFTRU5 |
|  |  |  |
| *Ardea cinerea* | Grey heron | BT7 |
|  |  |  |
| *Caracara plancus* | Crested caracara | CXPIP23 |
|  |  |  |
| *Ciconia ciconia* | White stork | GRW02 |
|  |  |  |
| *Columba vitiensis* | Metallic pigeon | GRW09 |
|  |  |  |
| *Crex crex* | Corn crake | GRW11 |
|  |  |  |
| *Cuculus canorus* | Common cuckoo | LINN1 |
|  |  |  |
| *Cyanistes caeruleus* | Eurasian blue tit | NEW1_PIsm |
|  |  |  |
| *Dendrocopos major* | Great spotted woodpecker | NEW3_Plsm |
|  |  |  |
| *Eurostopodus mystacalis* | White-throated nightjar | NYCNYC01 |
|  |  |  |
| *Phalacrocorax carbo* | Great cormorant | SGS1 |
|  |  |  |
| *Podiceps cristatus* | Great crested grebe | SW2 |
|  |  |  |
| *Psittacus erithacus* | Grey parrot | SYAT05 |
|  |  |  |
| *Quelea quelea* | Red-billed quelea | SYAT38 |
|  |  |  |
| *Sula leucogaster* | Brown booby | SYBOR10 |
|  |  |  |
| *Tetrastes bonasia* | Hazel grouse | TURDUS1 |
|  |  |  |
| *Turdus merula* | Common blackbird |  |
| *Upupa epops* | Eurasian hoopoe |  |
| *Urocolius indicus* | Red-faced mousebird |  |
