# Supplementary material for: Multiplex vs. singleplex assay for the simultaneous identification of the three components of avian malaria vector-borne disease by DNA metabarcoding
Source: PeerJ. 2025 Mar 18;13:e19107. doi: 10.7717/peerj.19107 (PMC11927560; doi:10.7717/peerj.19107)
Supplement: Table S2 [file peerj-13-19107-s003.docx]

**Table S2**. Primers used in this study.

| Target  taxa | Primer name | Primer sequence (5'-3') | Annealing temperature [°C] | Metabarcode  size [bp] | Reference |
| --- | --- | --- | --- | --- | --- |
| Aves | *Aves02_F* | GAAAATGTAGCCCATTTCTTCC | 56 | 66-86 | [(Taberlet et al., 2018)](https://www.zotero.org/google-docs/?vcW8Gr) |
|  | *Aves02_Cyan_F* | GAAAATGTAGCCCATTACTGCC |  |  | This study, modified from [(Taberlet et al., 2018)](https://www.zotero.org/google-docs/?ls6HTV) |
|  | *Aves02_Parus_F* | GAAAATGTAGCCCATTGCTGCC |  |  | This study, modified from [(Taberlet et al., 2018)](https://www.zotero.org/google-docs/?G365N9) |
|  | *Aves02_R* | CATACCGCCGTCGCCAG |  |  | [(Taberlet et al., 2018)](https://www.zotero.org/google-docs/?8sD3VB) |
|  | *Vert01_R* | TAGAACAGGCTCCTCTAG |  |  | [(Riaz et al., 2011)](https://www.zotero.org/google-docs/?zWFhtX) |
| Culicidae | *Culi01_F* | ACGCTGTTATCCCTAAGGTAACTTA | 60 | 144-147 | [(Schneider et al., 2016)](https://www.zotero.org/google-docs/?UtH1sM) |
|  | *Culi01_R* | GACGAGAAGACCCTATAGATCTTTAT |  |  | [(Schneider et al., 2016)](https://www.zotero.org/google-docs/?nhyBm1) |
| *Plasmodium* | *Plas01_F* | TATGGGATAWTTGTARTACACC | 55 | 37-44 | This study |
|  | *Plas01_R* | CCAGGCATGCAATACCGA |  |  | This study |
