# Supplementary material for: Multiplex vs. singleplex assay for the simultaneous identification of the three components of avian malaria vector-borne disease by DNA metabarcoding
Source: PeerJ. 2025 Mar 18;13:e19107. doi: 10.7717/peerj.19107 (PMC11927560; doi:10.7717/peerj.19107)
Supplement: Table S3 — Concentration corresponds to the final concentration in the PCR mix. Lineage corresponds to Plasmodium mtDNA cytochrome b lineage (Bensch et al., 2009). [file peerj-13-19107-s004.docx]

**Table S3**. Positive controls used for each primer pair. Concentration corresponds to the final concentration in the PCR mix. Lineage corresponds to *Plasmodium* mtDNA cytochrome *b* lineage [(Bensch et al., 2009)](https://www.zotero.org/google-docs/?6ZdU0Z).

|  | Species | Concentration (ng/µl) |
| --- | --- | --- |
| C+ *Culi01* | *Aedes koreicus* | 2 |
|  | *Aedes japonicus* | 2 |
| C+ *Aves02_mix* | *Psittacus erithacus* | 0.148 |
|  | *Neopsephotus bourkii* | 0.148 |
|  | *Quelea quelea* | 0.148 |
|  | *Urocolius indicus* | 0.148 |
| C+ *Plas01* | Lineage AFR046 | NA |
|  | Lineage AEDVEX01 | NA |
