# Supplementary material for: Multiplex vs. singleplex assay for the simultaneous identification of the three components of avian malaria vector-borne disease by DNA metabarcoding
Source: PeerJ. 2025 Mar 18;13:e19107. doi: 10.7717/peerj.19107 (PMC11927560; doi:10.7717/peerj.19107)
Supplement: Table S4 — Samples which co-amplified a mammal and an avian species were considered as potential mammal DNA contamination and retained on the basis of their avian hosts. Notice low overall performance of Vert01 to identify the avian host, compared to Aves02_mix. [file peerj-13-19107-s005.docx]

**Table S4**. Results of the *Vert01* amplification on the samples. Samples which co-amplified a mammal and an avian species were considered as potential mammal DNA contamination and retained on the basis of their avian hosts. Notice low overall performance of *Vert01* to identify the avian host, compared to *Aves02_mix*.

| Sample | Host (*Vert01*) | Non-avian host |
| --- | --- | --- |
| 1Da | NA | No |
| 2Da | NA | No |
| 3Da | Passeriformes | No |
| 4Da | NA | No |
| 5Da | NA | No |
| 6Da | Passeriformes ; *Homo sapiens* | No |
| 7Da | Passeriformes | No |
| 8Da | NA | No |
| 9Da | NA | No |
| 10Da | NA | No |
| 11Da | NA | No |
| 12Da | Passeriformes | No |
| 13Da | *Muscicapa striata* | No |
| 14Da | NA | No |
| 15Da | Passeriformes | No |
| 16Da | *Sus scrofa* | Yes |
| 17Da | Passeriformes | No |
| 18Da | NA | No |
| 19Da | NA | No |
| 20Da | NA | No |
| 21Da | Columbidae | No |
| 22Da | Passeriformes | No |
| 23Da | *Apodemus sp.* | Yes |
| 24Da | NA | No |
| 25Da | Columbidae | No |
| 26Da | Passeriformes | No |
| 27Da | *Apodemus sp.* | Yes |
| 28Da | Passeriformes | No |
| 29Da | Passeriformes ; Sus scrofa | No |
| 1Ma | *Capreolus capreolus* | Yes |
| 2Ma | *Equus sp.* | Yes |
| 3Ma | NA | No |
| 4Ma | *Henicorhina leucosticta* | No |
| 5Ma | *Buteo buteo* | No |
| 6Ma | Passeriformes ; *Sus scrofa* | No |
| 7Ma | Passeriformes ; *Sus scrofa* | No |
| 1a | Passeriformes ; *Sus scrofa* | No |
| 2a | Passeriformes | No |
| 3a | NA | No |
| 4a | Passeriformes | No |
| 5a | Passeriformes | No |
| 6a | NA | No |
| 7a | NA | No |
| 8a | NA | No |
| 9a | NA | No |
| 10a | NA | No |
| 11a | NA | No |
| 12a | NA | No |
| 13a | NA | No |
| 14a | NA | No |
| 15a | NA | No |
